# Mycotic aneurysm of the inferior gluteal artery caused by non-typhi *Salmonella *in a man infected with HIV: a case report

**DOI:** 10.1186/1752-1947-4-273

**Published:** 2010-08-18

**Authors:** Jon Fielder, Kenneth Miriti, Peter Bird

**Affiliations:** 1 Partners in Hope, PO Box 302, Lilongwe, Malawi; 2 University of Maryland, Institute of Human Virology, PO Box 495-00606, Nairobi, Kenya; 3 AIC Kijabe Hospital, PO Box 20, Kijabe 00220, Kenya

## Abstract

**Introduction:**

Non-typhi *Salmonellae *infections represent major opportunistic pathogens affecting human immunodeficiency virus-infected individuals residing in sub-Saharan Africa. To the best of our knowledge, we report the first documented case in the medical literature of a *Salmonella*-induced mycotic aneurysm involving an artery supplying the gluteal region.

**Case presentation:**

A 37-year-old black, Kenyan man, infected with human immunodeficiency virus with a CD4 count of 132 cells per microliter presented with a pulsatile gluteal mass and debilitating pain progressing over one week. He was receiving prophylaxis with trimethoprim-sulfamethoxazole. Aspiration of the mass yielded gross blood. An ultrasound examination revealed a 37 ml vascular structure with an intra-luminal clot. Upon exploration, a true aneurysm of the inferior gluteal artery was identified and successfully resected. A culture of the aspirate grew a non-typhi *Salmonellae *species. Following resection, he was treated with oral ciprofloxacin for 10 weeks. He later began anti-retroviral therapy. Forty-two months after the initial diagnosis, he remained alive and well.

**Conclusions:**

Clinicians caring for patients infected with human immunodeficiency virus in Africa and other resource-limited settings should be aware of the invasive nature of *Salmonella *infections and the potential for aneurysm formation in unlikely anatomical locations. Rapid initiation of appropriate anti-microbial chemotherapy and surgical referral is needed. Use of trimethoprim-sulfamethoxazole prophylaxis does not routinely prevent invasive *Salmonella *infections.

## Introduction

Non-typhi *Salmonellae *(NTS) bacteremia was recognized early in the course of the human immunodeficiency virus (HIV) epidemic in Africa as a common and serious opportunistic infection [1]. These organisms continue to constitute a significant burden of disease in this population. NTS were the most common cause of bacteremia among patients admitted to a hospital in southern Malawi, and nearly all cases occurred in HIV-infected individuals [2]. Likewise, a series from Nairobi, Kenya found NTS to be the most frequently-isolated organisms in HIV-infected patients [3]. Case fatality and recurrence rates are high, even following appropriate therapy. In a series from Malawi, 47 percent of patients died in hospital, while 43 percent experienced at least one recurrence during the following six months [4].

Bacteremia results from the invasive capacity of NTS and can lead to widespread tissue seeding. Immunocompromised individuals, including those with HIV infection, are at a high risk of disseminated disease [5]. In the elderly and those with co-morbid conditions, endovascular infections with *Salmonellae *species primarily affect the aorta [6]. Rupture of a *Salmonella*-induced mycotic aneurysm of the femoral artery has been reported in the case of an HIV-infected patient [7]. We describe a mycotic aneurysm of the inferior gluteal artery caused by NTS occurring in an adult Kenyan man infected with HIV. To the best of our knowledge, this report represents the first of its kind in the medical literature.

## Case presentation

A 37-year-old black Kenyan man presented to our HIV clinic with a chief complaint of left buttock pain. The pain had begun one week prior and gradually progressed over several days. During the few days before presentation, the pain had become severe and radiated down the back of his left leg making ambulation difficult. The pain worsened upon sitting or application of pressure. Over-the-counter analgesics provided no relief. He also reported subjective fever. A review of systems was otherwise non-contributory.

His past medical history was significant due to a motor vehicle accident 15 years prior to presentation. He was thrown from the vehicle and landed on his left hip although no fracture resulted. He had been diagnosed with HIV infection two months before the current illness. His CD4 count at that time was 132 cells per microliter. Two weeks prior to presentation, he was treated for thrush and diarrhea with miconazole oral-adhesive tables and metronidazole, respectively. He denied previous surgeries, hospitalizations, or other major illnesses. He was using daily trimethoprim-sulfamethoxazole (80-400 mg) for prophylaxis of opportunistic infections. He denied any allergies to medication.

He lived in rural Kenya with his wife and three children, all of whom tested negative for HIV infection. He worked as a farmer and was previously employed as a bus driver. He smoked cigarettes for two years but stopped 16 years prior to admission. He used alcohol for 11 years but had recently stopped.

On physical examination, his vital signs were: temperature 37. 6°C, pulse rate 94 beats per minutes, blood pressure 140/70 mm/Hg, and weight 59 kilograms. He was in acute distress, secondary to severe left buttock pain. His sclerae were anicteric and there were no palpable lymph nodes. Examination of his heart and lungs was unremarkable. He had no skin rash. His abdomen was soft without tenderness or palpable masses.

Examination of his inferior left buttock revealed exquisite tenderness in a 3 by 3 cm area with an underlying mass appreciated. External skin mottling was present. A second examiner noted that the mass was pulsatile. The patient walked with great difficulty due to pain. His motor strength was 5/5 in both extremities. He had no sensation to light touch in his left posterior calf. His patellar deep-tendon reflexes were 2+ bilaterally. Ankle jerks could not be elicited bilaterally.

**Figure 1:**
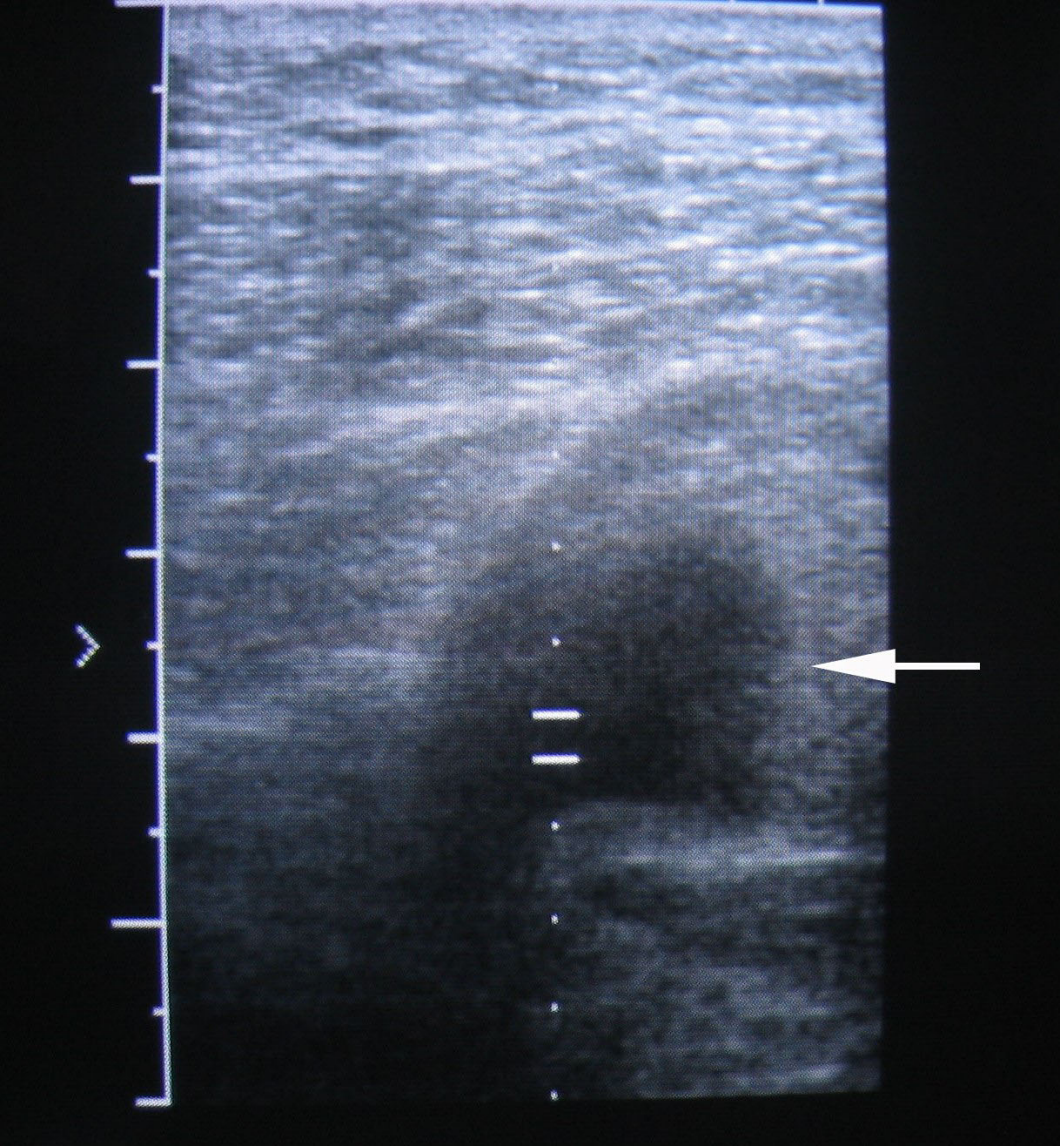
**An ultrasound examination of his left buttock performed on the day of presentation showing an unexpected wide-diameter, pulsatile vascular structure with intra-luminal clot (arrow)**.

The primary clinician attempted a percutaneous needle aspiration of a suspected abscess and obtained pure blood. A subsequent clinician noted the pulsatile nature of the mass and no further aspiration was attempted. An ultrasound examination of his left buttock demonstrated a vascular structure measuring 37 mm in diameter (Figure 1) with evidence of intra-luminal clot.

His hemoglobin was 12.9 g/dl. The bloody aspirate, obtained prior to the administration of antibiotics, was sent for culture. He was admitted to our hospital and begun on 2 g of cefazolin delivered intravenously every eight hours and 750 mg of ciprofloxacin delivered orally twice per day.

**Figure 2:**
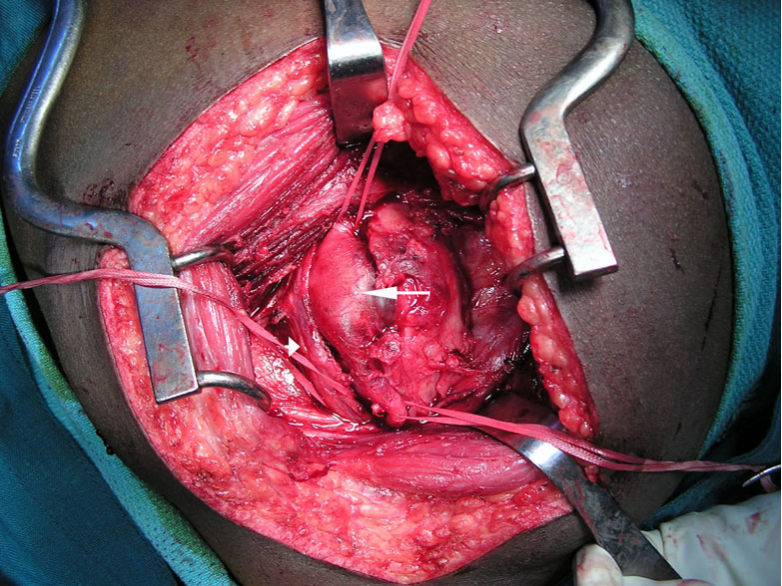
**Grossly-enlarged aneurysm of his inferior gluteal artery (arrow) compressing his sciatic nerve (arrowhead) found at our surgery the day following presentation**.

**Figure 3:**
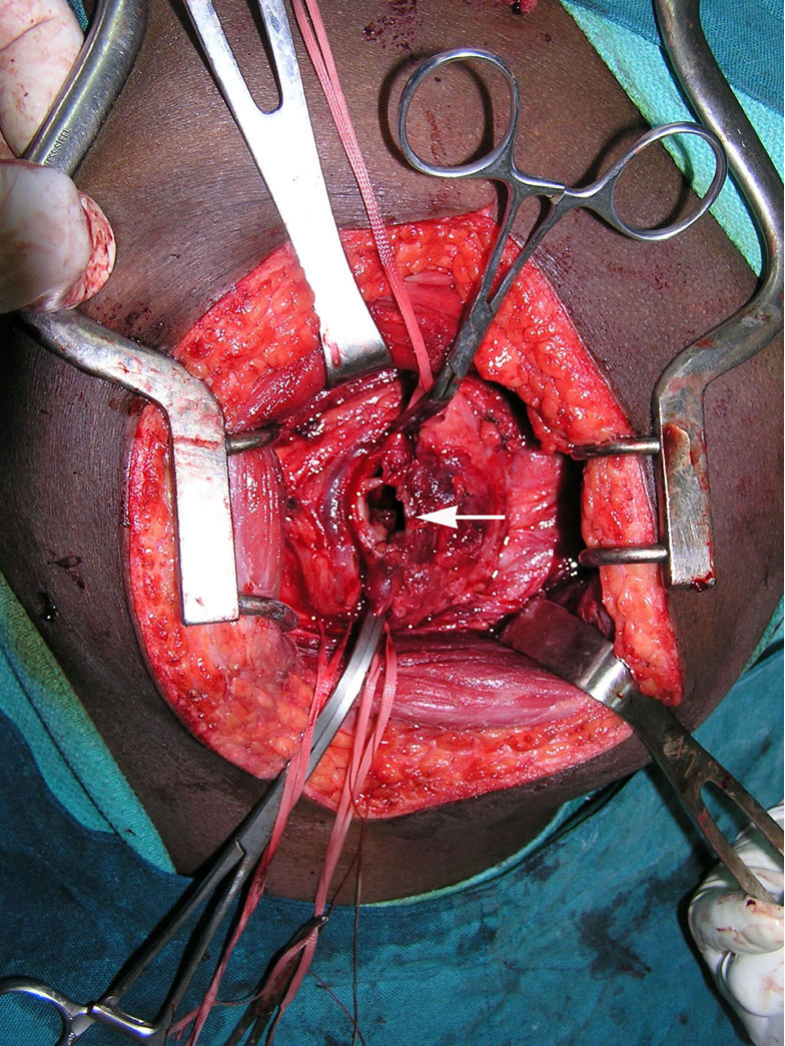
**End-on view (arrow) of true aneurysm of his inferior gluteal artery**.

The next morning, an exploration of his left buttock was performed under general anesthesia in the operating theater. A grossly-enlarged aneurysm of his inferior gluteal artery was discovered just below his piriformis muscle (Figures 2 and 3). The aneurysm had compressed his sciatic nerve. Dissection was difficult due to inflammation. Following proximal and distal ligation, the aneurysm was resected, with some wall left *in situ*. He tolerated the procedure well.

The aspirate was inoculated into a brain-heart infusion (BHI) broth and sub-cultured on to blood agar and MacConkey agar plates. The surgical specimen was not incubated for culture. Non-lactose fermenting Gram-negative rods were identified as NTS using a commercial kit (BioMerieux; Marcy l'Etoile, France). Further identification was not possible given our limited resources. The isolate was sensitive to tetracycline, gentamicin and kanamycin and resistant to ampicillin, chloramphenicol, trimethoprim-sulfamethoxazole, and streptomycin. We did not perform sensitivity testing to ciprofloxacin.

Two days after the operation, he was discharged home on 750 mg ciprofloxacin delivered orally twice daily. He completed 10 weeks of therapy. Two months after discharge, he began an anti-retroviral treatment with efavirenz, zidovudine, and lamivudine. Seven months later, his viral load was 966 copies per ml and his CD4 count had risen to 172 cells per microliter. Forty-two months after presentation, he was alive and had not experienced a recurrence of salmonellosis or of symptoms referable to the aneurysm.

## Discussion

To the best of our knowledge, this case is the first documented *Salmonella*-induced mycotic aneurysm affecting an artery supplying the buttock. The differential diagnosis of pulsatile gluteal masses is limited and includes aneurysms or pseudoaneurysms of the vessels feeding the gluteal region, including the inferior and superior gluteal arteries and a persistent sciatic artery [8]. Aneurysms may compress the sciatic nerve, producing pain and numbness as in our case report.

Combined surgical and medical treatment was indicated. The rapid development of severe symptoms in our case report suggested that rupture of the aneurysm was imminent. Inferior gluteal artery aneurysms may be resected followed by simple proximal and distal vessel ligation. Pulsatile lesions should not be aspirated. Although an aneurysm was not initially suspected in our case report, the pulsatile nature of the lesion should have first prompted an evaluation by ultrasound.

This isolate exhibited multi-drug resistance, a growing concern in sub-Saharan Africa [9]. Co-trimoxazole prophylaxis of opportunistic infections among HIV-infected individuals living in Uganda reduced morbidity, including diarrhea, and mortality despite the high prevalence of resistance to this agent [10]. However, co-trimoxazole use in our case report did not prevent invasive salmonellosis. Our hospital laboratory does not test for ciprofloxacin resistance, and the drug had only recently become widely available. Given the high cost of in-patient hospitalization for intravenous antibiotics, combined with successful removal of the endovascular source of infection, high-dose oral ciprofloxacin was administered for a prolonged period. Considering the significant rate of recurrence due to recrudescence reported in HIV-infected Africans, an extended course of antibiotics has been suggested as a way to reduce subsequent mortality.

Our hospital laboratory does not routinely incubate tissue specimens for culture. We cannot exclude the possibility that the aneurysm and the bacteremia were unrelated. The blood culture specimen was obtained by aspirating the lesion (which we do not recommend), but we cannot entirely rule out the possibility of incidental bacteremia. Incidental bacteremia could still have seeded an aneurysm produced by another cause. Given the rarity of aneurysms of the inferior gluteal artery, the lack of trauma, instrumentation, or another cause for the vascular lesion, and reports of *Salmonella *causing aneurysms in other large arteries [6,7], we believe NTS bacteremia is the most likely explanation for the presentation in this immunocompromised individual.

## Conclusions

Mycotic aneurysms should be considered in the differential diagnosis of pulsatile buttock lesions. Our case report indicates that NTS species are potential causative agents, particularly in immunocompromised patients living in areas marked by a high incidence of these infections. Clinicians caring for HIV-infected patients in Africa and other resource-limited settings should be aware of the invasive nature of *Salmonella *infections and the potential for aneurysm formation in unlikely anatomical locations. Such lesions should not be aspirated due to the risk of hemorrhage. Prompt surgical referral is required. A prolonged course of an appropriate antibiotic, taking into account the high rates of multi-drug resistance found among *Salmonella *species, should be considered due to the high risk of recrudescence and subsequent mortality. Prior use of trimethoprim-sulfamethoxazole prophylaxis does not rule out the possibility of invasive *Salmonella *infection.

## Competing interests

The authors declare that they have no competing interests.

## Authors' contributions

JF designed the case report form, conducted the literature review, was the major contributor in writing the manuscript, and supplied one of the figures. KM extracted all patient data from the medical chart and laboratory records. PB wrote the sections relating to the surgical intervention and supplied two of the figures. All authors participated in the review and discussion of the case, and all read, edited and approved the final manuscript.

## Consent

Written informed consent was obtained from the patient for publication of this case report and any accompanying images. A copy of the written consent is available for review by the Editor-in-Chief of this journal.
